# Perceptions of the Importance of Advance Care Planning During the COVID-19 Pandemic Among Older Adults Living With HIV

**DOI:** 10.3389/fpubh.2021.636786

**Published:** 2021-02-05

**Authors:** Annie L. Nguyen, Mariam Davtyan, Jeff Taylor, Christopher Christensen, Brandon Brown

**Affiliations:** ^1^Department of Family Medicine, Keck School of Medicine, University of Southern California, Los Angeles, CA, United States; ^2^Department of Pediatrics, Maternal, Child and Adolescent/Adult Center for Infectious Diseases and Virology, Keck School of Medicine, University of Southern California, Los Angeles, CA, United States; ^3^HIV + Aging Research Project-Palm Springs, Palm Springs, CA, United States; ^4^Department of Social Medicine, Population and Public Health, Riverside School of Medicine, University of California, Riverside, CA, United States

**Keywords:** advance directive, healthcare proxy, living will, POLST, ACP attitudes

## Abstract

**Background:** The importance of advance care planning (ACP) discussions have been heightened during the COVID-19 pandemic. We assessed advance directive completion, healthcare proxy (HCP), and attitudes toward ACP among older adults ages 50+ living with HIV during the COVID-19 pandemic.

**Methods:** Internet-based surveys were administered to 100 participants residing in the Coachella Valley, California from April to June 2020. We examined self-reported completion of an advance directive, HCP, and attitudes toward ACP before and after COVID-19. Adjusted regressions were performed on attitudes toward ACP.

**Results:** Participants' mean age was 64.2 years, most were non-Hispanic white (88.0%), men (96.0%), and identified as sexual minorities (96.0%). Many reported having an advance directive (59.6%) or HCP (67.3%). Most (57.6%) believed ACP to be more important now compared to the pre-pandemic era. Having an advance directive was associated with increase in age, higher education, living with other people, never having an AIDS diagnosis, and current undetectable viral load (*p* < 0.05). Having a HCP was associated with higher education, being married/partnered, and living with other people (*p* < 0.05). In a logistic regression model adjusted for education and living situation, the belief that ACP was more important during COVID was associated with not having an advance directive (OR: 5.07, 95% CI: 1.78–14.40) and fear of COVID-19 infection (OR: 4.17, 95% CI: 1.61–10.76.)

**Conclusions:** The COVID-19 pandemic presents a window of opportunity to engage people aging with HIV in ACP discussions, particularly those who do not already have an advance directive.

## Introduction

Advance care planning (ACP) describes a healthcare behavior where an individual engages in the process of articulating their values and preferences for end-of-life care so that their providers can make treatment decisions that align with their values (1). The ACP process typically involves discussions with loved ones or providers and many tools exist to assist with those discussions. Advance directives are legal documents that conveys healthcare wishes and are examples of one such planning tool. Advance directives typically fall into two broad types of documents. First, there are documents such as a living will, which specify desired treatment in the event the individual does not have the capacity to make decisions. Second, a durable power of attorney for healthcare or healthcare proxy (HCP) may specify wishes about healthcare but also legally appoints another person, the agent, to make decisions on behalf of the individual, the principal, in the event of incapacitation (2). There are other ACP tools, such as Physician Orders for Life-Sustaining Treatment (POLST), which are similar to advance directives in intent but are medical orders and not legal documents. A POLST may include an agent but does not require determination of incapacity of the principal for the medical orders to take effect.

ACP is complex and there may be variations in implementation of ACP tools across geographies, but the evidence for ACP is generally in support of its benefits to patients, families, and providers. Studies have shown that advance directives increase compliance with end-of-life wishes (3–5), improve the quality of communication between providers and decision-makers (4), and reduce caregiver stress (6). In a scoping review conducted by McMahan et al. (7), overall findings were positive in regards to ACP and health outcomes but some studies described mixed results on the impact of ACP on quality of life and healthcare utilization, indicating that there are limitations to the benefits of ACP. In a randomized trial of an ACP intervention, Sinclair et al. (8) found mixed results where participants in the ACP intervention arm experienced shorter stays in acute hospital settings and longer stays in palliative care settings compared to controls, but had no differences in other utilization outcomes such as emergency department visits and total hospital admissions. Another intervention study (9) using shared decision-making processes to integrate ACP into medical treatment orders, found no differences in overall mortality rates, in-hospital mortality rates, and length of hospital stays between the intervention and control groups but saw reductions in emergency visits and hospitalizations among the control group. Thus, the benefits of ACP may be more apparent in outcomes related to patient-centered values of communication and collaboration than in certain utilization outcomes.

People of all ages are encouraged to engage in ACP and complete advance directives but with greater emphasis for older adults. In the United States, the advance directive uptake is low and estimated that about one-third of adults have engaged in some form of ACP (10). Some clinicians have argued that the value of ACP has become more apparent during the COVID-19 pandemic (11). The novel coronavirus 2019 (COVID-19) pandemic can be characterized as a “focusing event”(12), that is, a critical moment that brings a particular issue to the forefront of the public psyche. In the wake of serious discussions about care rationing amidst healthcare resource scarcity, the pandemic has prompted healthcare providers to highlight the importance of engaging individuals in ACP discussions prior to the onset of serious acute illness or hospitalization (13, 14). The surge of hospitalizations prompted Block et al. (14) to call for a “massive upscaling of ACP” not just as a direct response to the pandemic but to better prepare for all end-of-life care. They reiterate the argument that clearly documenting end-of-life wishes can prevent situations where an unexpected, acute admission to an intensive care unit leaves family members ill-prepared to discuss end-of-life care. This reminder of the importance of ACP and call for its upscaling is timely. ACP is already recommended for all older adults and the urgency is amplified given that the risk for complications and mortality due to COVID-19 are associated with older age and underlying medical conditions (15, 16). There may already be some heightened public awareness and response to the importance of ACP as a result of the pandemic. One study showed a nearly 5-fold increase in online advance directive completions during the COVID-19 pandemic compared to the months leading up to the pandemic (17).

In this paper, we describe the results of an internet-based survey (18) that was implemented in the early months of the COVID-19 pandemic to assess the impact of the pandemic on older adults living with HIV. Conventionally defined among people living with HIV as ages 50 and over (19), older adults account for about half of all people living with HIV in the US (20). Older adults living with HIV are at increased risk for conditions that exacerbate COVID-19 complications, such as cardiovascular disease, diabetes, certain cancers, and multi-morbidity (21, 22) and fall into two risk categories for COVID-19-related hospitalizations based on older age and chronic conditions. Our analyses here describe (1) the factors associated with completion of advance directives among older adults living with HIV, and (2) the relationship between fear of getting COVID-19 and attitudes toward ACP.

## Methods

We conducted cross-sectional, internet-based surveys on Qualtrics with community-dwelling, older adults living with HIV in the Coachella Valley, CA. Participants were recruited through email distribution lists from local organizations, screened by a research coordinator over the phone for inclusion criteria (i.e., age 50 or over, identify as living with HIV, reside in the Coachella Valley area), and provided with a link to the survey. A consent page was presented for acknowledgment prior to the first page of the survey. Participants received a $10 e-gift card incentive after completing the survey, to an e-mail unlinked to their responses. All data were collected between May and July 2020. This study was reviewed and approved by the University of Southern California Institutional Review Board.

### Measures

Demographic information collected included age, race/ethnicity, education, gender identity, sexual orientation, employment status, relationship status, and living situation (alone vs. with other people). HIV characteristics collected included length of HIV diagnosis (years), ever had an AIDS diagnosis, current viral load (detectable vs. undetectable), and current CD4 count (<200, 200–500, >500 cells/mm^3^).

We collected data about ACP through self-report. Advance directive completion and HCP were collected via self-report. For the purposes of analyses, participants were categorized as having an advance directive if they answered “yes” to the following: “Do you currently have an advance directive, living will, or signed Physician Orders for Life-Sustaining Treatment (POLST) form?” Although POLST are not considered advance directives in the legal sense, we considered them in this variable because they are similar to advance directives in scope and intent. A separate variable identified participants who indicated they had a HCP: “Do you currently have a designated healthcare proxy, medical decision maker, or someone who is legally designated to make healthcare decisions for you?” To assess attitudes toward ACP, participants were asked “How has your thinking about advance care planning changed because of the COVID-19 pandemic?” Responses choices were “I think ACP is more important now compared to before,” “I think ACP is less important now compared to before, or “My thinking about ACP has not changed.”

Fear of getting COVID-19 was assessed from 1 item on the Pandemic Stress Index (PSI) (23) which includes a checklist of behaviors and stresses experienced (e.g., felt anxious, not getting enough financial support, change in sleep patterns, etc.) as a result of the COVID-19 pandemic. “Fear of getting COVID-19” was a response choice and selections were treated as a dichotomous variable (selected = yes vs. not selected = no) for analyses.

### Data Analyses

Descriptive statistics are reported for demographics variables. Bivariate comparisons to determine the differences between having an advance directive (yes vs. no) or HCP (yes vs. no) were performed using *t*-tests for continuous variables and chi-squared tests or Fisher's exact tests for categorical variables. Although there were three responses choices for the question on ACP attitudes, none of the participants believed ACP to be less important compared to before the pandemic. Thus, bivariate analyses compared the differences between participants who believed ACP to be more important vs. no change in attitude. Attitudes toward ACP was further probed with logistic regression analyses to test associations with belief that ACP is more important now compared to before the pandemic. Model 1 tested fear of getting COVID-19 as the only factor. Having an advance directive and HCP were both associated with attitudes toward ACP and were included in Model 2. However, because having an advance directive and an HCP were highly correlated, we combined these into one variable to indicate engagement in any ACP. In Model 3, we adjusted for living alone and education because these factors were consistently associated with having an advance directive or HCP. Critical alpha was set at 0.05.

## Results

Participant demographics are presented in Table 1. Briefly, the mean age of participants (*N* = 100) was 64.2 years (*SD* = 6.7, range: 51–86). The majority of participants were non-Hispanic white (88.0%), men (96.0%), gay or lesbian (93.0%), and completed some college education or higher (93.9%). The average number of years living with HIV was 26.9 (*SD* = 8.5, range: 5–39). The majority (54.5%) had received an AIDS diagnosis in their lifetime, had CD4 counts >500 cells/mm^3^ (58.2%), and reported undetectable viral loads (90.8%).

**Table 1:** Participant demographic and HIV characteristics (*N* = 100).

**Characteristics**	***n* (%)**
**Age, mean (SD)**	64.2 (6.7)
**Race/ethnicity**	
Black/African American	4 (4.0%)
Hispanic/Latino	6 (6.0%)
Non-Hispanic white	88 (88.0%)
More than one race	2 (2.0%)
**Gender identity**	
Man	96 (96.0%)
Woman	3 (3.0%)
Gender non-conforming	1 (1.0%)
**Sexual orientation**	
Gay or lesbian	93 (93.0%)
Straight or heterosexual	4 (4.0%)
Bisexual	2 (2.0%)
Other identity	1 (1.0%)
**Highest education achieved**	
High school graduate/GED	6 (6.1%)
Some college	29 (29.3%)
College graduate	35 (35.4%)
Post-college studies	29 (29.3%)
**Relationship status**	
Single, unpartnered	54 (54.0%)
Legally married/partnered	46 (46.0%)
**Living situation**	
Live alone	50 (50.0%)
Live with spouse/partner or others	50 (50.0%)
**Years living with HIV, mean (SD)**	26.9 (8.5)
**AIDS diagnosis ever (yes)**	54 (54.5%)
**Undetectable viral load (yes)**	89 (90.8%)
**Current CD4 count (cells/mm**^**3**^**)**	
<200	2 (2.2%)
200–500	34 (36.6%)
>500	57 (61.3%)

Many participants reported that they currently had an advance directive (59.6%) or an HCP (67.3%). More than half (57.6%) believed ACP to be more important now than compared to before the COVID-19 pandemic and the remainder stated that their attitude toward ACP had not changed. None of the participants indicated that ACP was less important now compared to before the pandemic.

In bivariate analyses, having an advance directive was associated with older age (65.3 vs. 62.6 years, *p* = 0.05), college graduate or greater education (76.3 vs. 47.7%, *p* = 0.001), living with other people (59.3 vs. 37.5%, *p* = 0.03), never been diagnosed with AIDS (50.9 vs. 60.0%, *p* = 0.03), and current undetectable viral load (96.6 vs. 84.2%, *p* = 0.05). Having an HCP was associated with college graduate or greater education (75.8 vs. 45.2%, *p* = 0.003), being married or partnered vs. single or not partnered (57.6 vs. 21.9%, *p* = 0.001), and living with other people (62.1 vs. 25.0%, *p* = 0.001). Compared to participants who reported no change in their attitudes toward ACP, those who believed it to be more important were less likely to have an advance directive (45.6 vs. 78.6%, *p* = 0.001) and less likely to have an HCP (58.9 vs. 78.6%, *p* = 0.04).

The results of the logistic models are presented in Table 2. In the univariate model, fear of getting COVID-19 (*p* = 0.005) was associated with greater odds of the belief that ACP was more important now. In Model 2, not engaging in any ACP (*p* = 0.01) and fear of getting COVID-19 (*p* = 0.004) were both associated with greater odds of believing that ACP was more important now. Finally, the adjusted model showed both factors (*p*'s < 0.05) remained associated with greater odds of believing that ACP was more important now.

**Table 2:** Logistic regression models predicting belief that advance care planning is more important now compared to before the COVID-19 pandemic.

**Variable**	**Model 1**			**Model 2**			**Model 3**		
	**B**	**SE**	**OR (CI)**	**B**	**SE**	**OR (CI)**	**B**	**SE**	**OR (CI)**
**Fear getting COVID-19**	0.61^**^	0.22	3.38 (1.44–7.94)	0.67^**^	0.23	3.84 (1.56–9.48)	0.70^**^	0.23	4.09 (1.68–10.58)
**Do not any ACP**	–	–	–	0.61^*^	0.25	3.38 (1.25–9.15)	0.61^*^	0.28	3.36 (1.13–11.11)
**Live alone**	–	–	–	–	–	–	−0.01	0.24	0.97 (0.38–2.48)
**College or greater education**	–	–	–	–	–	–	0.09	0.25	1.19 (0.45–3.20)

* *< 0.05*,

** *< 0.01*.

## Discussion

The COVID-19 pandemic has prompted urgent calls from clinicians and researchers globally for coordinated efforts to increase ACP uptake (14, 24–26). Engaging in ACP well in advance of acute hospitalization better prepares the patient and their loved ones for conversations with providers in acute care settings so that they can make the best care decisions (14).

The findings from our study show that most of our participants had conveyed their end-of-life wishes in a document such as an advance directive, living will, or POLST (59.6%) or a healthcare proxy (67.3%). These rates are higher than reflected in the literature for people living with HIV among whom advance directive completion rates range from 8 to 47% (27–29). Because our study was conducted in the first few months of the COVID-19 pandemic, it is possible that the heightened discussions of hospitalization and mortality in the media prompted participants to engage in ACP. Our survey did not assess the timeframe for when these tools were completed so we are unable to extrapolate whether the high rates of engagement in ACP were already present before the pandemic began. Our participants lived in the Palm Springs, California region, which is a well-established retirement community. As such, it is possible that participants have greater access to local resources that cater to the specific needs of older adults, including end-of-life planning.

Our findings also show that more than half of the participants in this study believe ACP to be more important now compared to the time before the COVID-19 pandemic. Although 42.4% stated that their thinking about ACP had not changed, no one thought that it was less important. Multivariate models suggest that people who do not already engage in ACP and people who are fearful of COVID-19 have greater odds of believing that ACP is more important now. These findings indicate that there may be a window of opportunity during and after the COVID-19 pandemic to engage community-dwelling older adults living with HIV in ACP if they have not already done so. Fried et al. (30) assert that ACP should be understood as a process of health behavior change because there is large variation in an individual's degree of readiness to engage in ACP. According to the Transtheoretical Model, behavior change can be described through temporal dimensions in terms of the five stages of readiness for change: (1) pre-contemplation, (2) contemplation, (3) preparation, (4) action, and (5) maintenance (31). Interventions aim to move individuals toward the “action” and “maintenance” end of the continuum. Extrapolating these concepts to our study findings suggest that some older adults living with HIV who do not already engage in ACP may be more ready to act as a result of the pandemic. From an intervention perspective, the discussions about end-of-life care that have been raised during the COVID-19 pandemic may act as a starting point to engage older adults living with HIV in conversations about ACP.

From a public health policy perspective, the pandemic as a “focusing event” may offer an opportunity to promote the importance of ACP and prompt older adults along the ACP behavior change continuum through population-based efforts. For example, researchers have put forth recommendations for health systems to implement ACP interventions such as mass dissemination of materials to prepare individuals to engage in ACP discussion (14). Health systems can create and deliver ACP promotion messages in collaboration with local groups and communities, such as senior centers, LGBT resource centers, or HIV-related service or social groups, to be more relevant to specific populations. Community-based strategies can also tap into local strengths and resources for greater reach. Specifically in Palm Springs, Planning Ahead for LGBT Seniors (PALS) is a community initiative that offers informational seminars and social-based gatherings for discussions about planning ahead for health-related events like end-of-life care. These types of community partners may be helpful in activating the kind of “massive upscaling of ACP” called for by Block et al. (14).

In addition to some of the limitations already noted, our participant sample was primarily white, male, and college educated and findings may not be generalizable to other demographic groups. It is documented in the literature that ethnic minorities have lower rates of advance directive completion compared to whites among people living with HIV (32). Thus, are other factors that may need to be considered in ACP among ethnic minorities that were not included in our study. Scaling up efforts to increase uptake of ACP across all populations will require examination of the root causes of the barriers toward ACP. It is also worth noting that we used a functional definition of advance directives in our analyses and grouped POLST forms together with living wills and advance directive documents while separating healthcare proxy as a separate variable. This functional definition allowed us to differentiate between the completion of documents that specify desires for end-of-life care procedures (e.g., resuscitation, tube feeding, mechanical ventilation, etc.) and completion of documents that designate another person to make healthcare decisions for the individual. This distinction may not be completely congruent with the definition of advance directives in the literature so caution is urged in interpreting findings.

The COVID-19 pandemic has challenged the healthcare system in many unexpected ways and has prompted a call for greater engagement in ACP. Completing advance directives and HCPs are just two of the many ways to engage in discussions about ACP. Because many older adults living with HIV fall into two risk categories for COVID-19 complications and mortality, older age and having chronic comorbidities, the importance of ACP is elevated. Our study findings show that among a group of older adults living with HIV in Palm Springs there is room for improvement for ACP engagement and an opportunity to do so while the pandemic has heightened conversations around ACP.

## Data Availability Statement

The datasets presented in this article are not readily available because Data sharing requests were not explicitly approved by the IRB. Requests to access the datasets should be directed to annie.nguyen@med.usc.edu.

## Ethics Statement

The studies involving human participants were reviewed and approved by University of Southern California Institutional Review Board. Written informed consent for participation was not required for this study in accordance with the national legislation and the institutional requirements.

## Author Contributions

The study was conceived by JT and AN with input from CC, BB, and MD. CC and JT assisted with data collection. AN performed the data analyses. The writing of the manuscript was led by AN with input from MD, JT, CC, and BB. All authors contributed to data interpretation and approved the final version.

## Conflict of Interest

The authors declare that the research was conducted in the absence of any commercial or financial relationships that could be construed as a potential conflict of interest.
